# *Nicotiana tabacum* as a dead-end trap for adult *Diaphorina citri*: A potential biological tactic for protecting citrus orchards

**DOI:** 10.3389/fpls.2022.1081663

**Published:** 2023-01-06

**Authors:** Lixia Zheng, Qianqian Xu, Gu Gong, Yonglin Liao, Min Yu, Sergey Shabala, Wensheng Chen, Weijian Wu

**Affiliations:** ^1^ International Research Center for Environmental Membrane Biology and Department of Horticulture, Foshan University, Foshan, China; ^2^ Laboratory of Insect Ecology, South China Agricultural University, Guangzhou, China; ^3^ Guangdong Provincial Key Laboratory of High Technology for Plant Protection, Plant Protection Institute, Guangdong Academy of Agricultural Sciences, Guangzhou, China

**Keywords:** Asian citrus psyllid, *Nicotiana tabacum*, Huanglongbing, nonhost plant, EPG, L-nicotine

## Abstract

The Asian citrus psyllid, *Diaphorina citri* Kuwayama (Hemiptera: Liviidae), is a key vector of the causal agents of Huanglongbing (HLB), a devastating disease affecting citrus almost worldwide. *Nicotiana tabacum* L. is an important commercial crop in China. Field observations suggested that *D. citri* adults die on *N. tabacum* leaves when grown nearby citrus orchards. In this study, the preference for and survivorship of *D. citri* adults on *N. tabacum* and their feeding behavior were investigated. The results showed that *D. citri* adults were attracted to *N. tabacum* and to the green leaf volatiles (GLVs) (*Z*)-3-hexenol and (*E*)-2-hexenol. The survival of *D. citri* adults on *N. tabacum* was less than 30 h, which was shorter than that for adults without food (35 h) and on a suitable host *Murraya exotica* L. (29 days). Electrical penetration graph (EPG) recordings revealed that the pathway phase of *D. citri* on *N. tabacum* leaves consisted of four waveforms—the non-probing phase (NP), the pathway phase (PP, including intercellular probing of activity in the phloem (C) and phloem penetration (D)), phloem salivation (E1), and phloem ingestion (E2). *Diaphorina citri* only secreted saliva and ingested sap from phloem on *N. tabacum* leaves and spent the longest duration in phloem sap ingestion (E2). Moreover, L-nicotine, an important defense compound against insects in *N. tabacum* plants, was highly toxic to *D. citri*. These results suggested that *N. tabacum* plants could help to sustainably control the spread of *D. citri* and HLB when growing in and around citrus orchards.

## Introduction

Huanglongbing (HLB) is a disease caused by the phloem-inhabiting bacterium *Candidatus* Liberibacter asiaticus (CLas) or *Ca.* L. americanus (CLam) (Bové, 2006). It is the most devastating citrus disease and a worldwide threat to the citrus industry (Bové, 2006; da Graça et al., 2016) because of the lack of a cure or/and an effective control method (Chin et al., 2014). The most important and effective measure to control the spread of HLB in citrus is the management of the key vector, the Asian citrus psyllid, *Diaphorina citri* Kuwayama (Hemiptera: Liviidae) (Stansly et al., 2014; Qureshi et al., 2014; Monzo and Stansly, 2015). *Diaphorina citri* nymphs and adults can acquire CLas by feeding on infected plants (Inoue et al., 2009; Pelz-Stelinski et al., 2010). If the pathogen is acquired at the nymphal stage, adults are able to transmit it immediately after emergence (Inoue et al., 2009). Previous studies have demonstrated that feeding on infected plants could increase the fecundity and produce more offspring of *D. citri* (Pelz-Stelinski et al., 2010; Pelz-Stelinski and Killiny, 2016). *Diaphorina citri* has highly efficient CLas transmission, and even an individual adult can successfully transmit the pathogen to healthy plants within a short time period (Pelz-Stelinski et al., 2010). Currently, *D. citri* and HLB have spread to almost all citrus-growing regions worldwide (Ghanim et al., 2017).

*Diaphorina citri* is an oligophagous insect with a host range restricted to plants in the Rutaceae family (Li, 2011) that includes more than 50 species (Halbert and Manjunath, 2004). However, *D. citri* also tends to feed on weeds of non-Rutaceae surrounding citrus orchards, such as *Bidens alba* L. (Asteraceae), *Eupatorium capillifolium* (Lam.) Small ex Porter & Britton (Asteraceae), *Ludwigia octovalvis* (Jacq.) P.H. Raven (Onagraceae) (Johnston et al., 2019; George et al., 2020), *Solanum nigrum* L. (Solanaceae), *Ageratum conyzoides* L. (Asteraceae), and *Praxelis clematidea* R.M. King & Robinson (Asteraceae) (Lu et al., 2021). These weeds have been suggested as alternative or secondary host plants for *D. citri* and allow the insect to survive in the absence of suitable hosts, although they are unable to reproduce and survive for an extended period on these hosts (Tiwari et al., 2010; Johnston et al., 2018; Johnston et al., 2019). Thus, these alternative hosts could play an important role in area-wide management of *D. citri* and affect control measures such as insecticide applications. *Diaphorina citri* can utilize hosts in the Rutaceae, Asteraceae, Onagraceae, Solanaceae, Moraceae (Thomas and de León, 2011; Felisberto et al., 2019), and Boraginaceae (Arshad et al., 2019), suggesting that their olfactory system can sense chemical signals emitted by these hosts. It also suggests that these signals might not be specific but common to several plant species such as green leaf volatiles (GLVs) (Riddiford, 1967).

GLVs are a series of six-carbon aldehydes and alcohols, their esters emitted by plants and produced from polyunsaturated fatty acids through the lipoxygenase (LOX) pathway (Holopainen and Gershenzon, 2010), such as (*Z*)-3-hexenol, 1-hexanol, (*Z*)-3-hexenyl acetate, and (±)-2-hexanol (Frati et al., 2008; Zheng et al., 2014). They usually serve as signal molecules to herbivorous insects in host finding. Although *D. citri* has a relatively simple olfactory system (Onagbola et al., 2008; Zheng et al., 2020), there are rhinarial plates known as the principal odorant sensors (Park and Hardie, 2002; Couthinho-Abreu et al., 2014) found on their antennae (Onagbola et al., 2008; Zheng et al., 2020), containing plant volatile-sensing olfactory neurons (Chapman, 1982; Kristoffersen et al., 2008). Couthinho-Abreu et al., 2014 demonstrated that the antennal neurons in *D. citri* strongly respond to odorants found in citrus. Several studies with *D. citri* revealed that stimuli emitted by flushing shoots of the host are implicated in host location (Patt and Sétamou, 2010; Sétamou et al., 2012; Sule et al., 2012). Therefore, these studies point to the feasibility of developing an odorant-based approach for improving *D. citri* surveillance and control.

*Nicotiana tabacum* is an important commercial crop in China that serves as a natural source of active compounds with important applications in medicine and agriculture for centuries (Peng et al., 2022). For instance, nicotine (a natural compound from *N. tabacum*) and its synthetic derivatives were used as insecticides against sucking insect pests (Feyereisen, 2018). Fu et al. (2008) found that (*E*)-2-hexenal and (*Z*)-3-hexenyl acetate (GLVs from *N. tabacum*) significantly attracted *Helicoverpa assulta* Guenée and *H. armigera* Hübner to *N. tabacum* plants. Several studies have demonstrated that *D. citri* adults may move to surrounding non-host plants (at least temporarily) when host conditions are unfavorable and insecticide sprays induce their dispersal (Lewis-Rosenblum et al., 2015; Johnston et al., 2018; Johnston et al., 2019; Lu et al., 2021). In South China, it is a common practice to plant *N. tabacum* around citrus orchards, and our field survey found that *D. citri* adults on *N. tabacum* leaves that moved from nearby citrus orchards were all dead (**Supplementary Figure 1**). While the applied significance of this finding for *D. citri* and HLB management can be significant, the mechanistic basis of this process remains unclear. We hypothesized that the GLVs from *N. tabacum* plants are involved in *D. citri* host location, especially when adults move to *N. tabacum* plants due to unfavorable host conditions. Following, adults on *N. tabacum* are killed by the plant’s secondary metabolites such as nicotine. Therefore, the main objective of this study was to determine the preference and performance of *D. citri* on *N. tabacum* that could offer some practical methods to control the spread of *D. citri* and HLB.

## Materials and methods

### Insects

Adult *D. citri* were collected from a colony established in 2019 at Foshan University that was initiated from insects collected from disease-free, ornamental plants of *Murraya exotica* L. (Rutaceae) in South China Agriculture University. They were kept without exposure to insecticides in a nylon net room with dimensions of 2.5 m (length, *L*) × 1.5 m (width, *W*) × 2.5 m (height, *H*) at 28 ± 1°C, 70 ± 5% relative humidity, and 14:10 L:D photoperiod.

### Plants

The plants used for the trials were potted seedlings. For the behavioral and survivorship assays and electrical penetration graph (EPG) experiments, potted seedlings of 3-year-old *M. exotica* (~40 cm) and *N. tabacum* (~30 cm, Yunyan 87) were used. They were grown with nutrient soil in a temperature-controlled greenhouse (28 ± 1°C) under natural sunlight conditions. Selected plants were washed and watered 24 h prior to the experiments.

### Chemicals

According to Yi et al. (2017), the main GLVs from *N. tabacum* are (*Z*)-3-hexenal (CAS: 929-96-1, purity of 98%), (*E*)-2-hexenol (CAS: 928-95-0, purity of 97%), (*E*)-2-hexenal (CAS: 85761-70-2, purity of 98%), (*Z*)-3-hexenyl acetate (CAS: 3681-71-8, purity of 98%), and (*E*)-2-hexenyl acetate (CAS: 2497-18-9, purity of 98%), and all were purchased from Sigma-Aldrich (Shanghai, China). These chemicals were tested individually in a Y-tube experiment.

### Behavioral response of *D. citri* adults

An H-tube olfactometer was used to test the preference of adult psyllids to *N. tabacum* plants. The H-tube olfactometer (**Supplementary Figure 2**) in this study was modified according to Khan and Saxena (1986). The H-tube olfactometer was made of odorless polypropylene plastic and consisted of two cylinders (arms) that were connected to a transverse tube (10-cm diameter, 30-cm long with a hole of 1 cm in the middle for releasing psyllids). The entire olfactometer was covered with an opaque cloth to protect psyllids from light. The *N. tabacum* plants served as an odor source, whereas the other (empty) arm was used as a control. Thereafter, starved adult psyllids were introduced individually in the middle of the transverse tube. If the tested individual crossed more than one-third of the choice area within 30 min after introduction, it was recorded as a positive response to the treatment or the control. Failure to move within this timeframe was recorded as no response. After 10 insects were tested, the samples at the two sides were exchanged to randomize any positional effects, and the H-tube olfactometer was washed with 75% alcohol. Each psyllid was tested only once, and 100 female and male *D. citri* were tested for each choice test. The adult psyllids were placed in glass tubes and deprived of food for 4 h prior to the experiments. Bioassays were performed under controlled conditions at 28 ± 1°C, 70 ± 5% relative humidity.

A Y-tube glass olfactometer was used to test the preferences of adult psyllids to the main GLVs from *N. tabacum*. The Y-tube olfactometer consisted of a 30-cm-long, 3-cm-diameter central tube and two 15-cm-long, 2-cm-diameter lateral arms, which were individually connected to the odor stimuli and a control through a Teflon connection. An atmospheric sample collector (model QC-1S, Beijing Labor Protection Institute Co. Ltd., Beijing, China) was used to draw the air to the apparatus. Air from the atmospheric sample collector was divided into two ways and passed through charcoal filters and humidifiers. Then, the humidified and purified air at 160 ml/min was introduced into the odor source bottles and each arm of the olfactometer (**Supplementary Figure 3**). The Y-tube was also covered with an opaque cloth to protect psyllids from light. In the tests, a 20-µl liquid paraffin applied on a filter paper (20 mm in diameter) was used as control, and 20 µl of the diluted test odor (20 µl/ml) applied on a filter paper (20 mm in diameter) served as an odor source. The test method and environment were the same as in the H-tube olfactometer assays.

### Survival duration of *D. citri* adults

The young twigs of *N. tabacum* and *M. exotica* potted plants were covered with an 80-mesh nylon net, and 20 (3–6-day-old) mated *D. citri* females and males were released into the net. Each *N. tabacum* potted plant was a replicate. Adults released in an empty box with a mesh cover were treated as the negative control (starvation treatment), and *M. exotica* plants were used as a positive control. The mortality of *D. citri* adults in each treatment was observed and recorded once a day until they were all dead. In addition, the number of eggs laid on *N. tabacum* and *M. exotica* leaves was also recorded once a day until all adults were dead. The trials were conducted at 28 ± 1°C, 70 ± 5% relative humidity. There were six replicates for each treatment.

### EPG analysis

A Giga-8 DC EPG system (Wageningen, the Netherlands) (Tjallingii, 1978) was used to record the feeding activities of adult *D. citri* on *N. tabacum* leaves. The *D. citri* adults were fixed using a negative pressure device. One end of a 3-cm length of gold wire (12.5-μm diameter) was connected to an amplifier through the EPG probe. The other end was connected to the dorsum of the *D. citri* adults with conductive silver glue (Wageningen Agricultural University). To complete the circuit, a copper electrode (10-cm length, 2-mm diameter) was inserted into the soil of the *N. tabacum* plants. There was a 30-min starvation period before the adult *D. citri* was placed on *N. tabacum* plants. All EPG experiments were recorded for 6 h and conducted inside a Faraday cage under 28 ± 1°C, 70 ± 5% relative humidity in a climate-controlled room. The EPG data were analyzed using EPG Stylet + software (Wageningen Agricultural University, 2012), and the waveforms recorded for *D. citri* probing behavior were characterized according to prior histological studies (Bonani et al., 2010; Yang et al., 2011; George et al., 2017). Windows Dataq Waveform Browser (Dataq Instruments Inc., Akron, OH, USA) was used to annotate waveforms. For the purposes of this study, five main feeding phases were distinguished by EPG, which were described based on their morphology (amplitude, frequency) and electrical (voltage level, electrical origin) characteristics and their correlations with stylet activities in the plant tissues (**Supplementary Figure 4**): (1) the non-probing phase (NP), (2) the pathway phase (PP, including intercellular probing of activity in the phloem (C) and phloem penetration (D)), (3) phloem salivation (E1), (4) phloem ingestion (E2), and (5) xylem ingestion (G) (Bonani et al., 2010; Yang et al., 2011; George et al., 2017). A total of 20 adult psyllids (10 males and 10 females) were recorded feeding on the *N. tabacum* potted plant.

### Toxicological bioassays

The toxicity of L-nicotine to psyllids was tested using a leaf-dip method. L-Nicotine (CAS: 54-11-5, purity of 98.57%) was supplied by Beijing Putian Tongchuang Biotechnology Co., Ltd. (Beijing, China). Stock solutions (250, 500, 1,000, 1,500, 2,000, and 4,000 mg/L) of L-nicotine were prepared with acetone. Young citrus leaves were collected and dipped into the test solutions for 10 s and dried by natural air ventilation. The leaves, with the leaf petiole covered with moist cotton, were placed into Petri dishes containing moistened filter papers to avoid desiccation of the leaves. Mock controls used citrus leaves treated with acetone only. Thirty adults were introduced into each Petri dish for a total of three Petri dishes (replicates) per L-nicotine concentration and held at 28 ± 1°C, 70 ± 5% relative humidity, and a photoperiod of 14:10 h (L:D). The mortality of *D. citri* adults in each treatment was observed and recorded after 12, 24, 48, and 72 h of exposure.

### Statistical analyses

Statistical analysis was performed using IBM SPSS Statistics 25.0. The H-tube and Y-tube olfactometer data were analyzed with the likelihood ratio statistic test (Kleinbaum and Klein, 2010). The survival rate of adult psyllids feeding on *N. tabacum* leaves was calculated using Kaplan–Meier’s survival analysis and was subjected to log-rank (Mantel–Cox) coefficient. EPG data were subjected to analysis of variance (ANOVA), and means were compared by the least significant difference (LSD) test. For toxicological bioassays, the corrected mortality rate (M_C_) of each treatment was calculated by the Abbot formula M_C_ = (M_2_-M_1_)/(1-M_1_) × 100, where M_1_ is the control mortality and M_2_ is the mortality of each treatment. LC_50_ was estimated using Probit analysis (Bliss, 1934).

## Results

### Behavioral response of adult *D. citri* to *N. tabacum* and its main GLVs

*Diaphorina citri* females and males were attracted to *N. tabacum* plants, (*Z*)-3-hexenol, and (*E*)-2-hexenol compared with the control (all *P* < 0.05) (**Table 1**). In addition, males were attracted to (*E*)-2-hexenal and (*Z*)-3-hexenyl acetate (**Table 1**). There were no significant differences between the numbers of female *D. citri* adults choosing (*E*)-2-hexenal, (*Z*)-3-hexenyl acetate, and (*E*)-2-hexenyl acetate and male *D. citri* adults choosing (*E*)-2-hexenyl acetate when compared with the control (all *P* > 0.05) (**Table 1**).

**Table 1:** Responses of *Diaphorina citri* adults to tobacco plant and synthetic standards of green leaf volatiles (GLVs).

Source of volatiles	# Response				Likelihood ratio statistic Λ(*x*)		*p*-value	
	Females		Males					
	Volatiles	Control	Volatiles	Control	Females	Males	Females	Males
Living plant	85	15	69	31	23.4900	6.4316	1.25E-06	0.0112
(*Z*)-3-Hexenol	80	20	90	10	16.7416	31.9697	4.28E-05	1.57E-08
(*E*)-2-Hexenol	92	8	84	16	35.9924	22.0168	1.98E-09	2.70E-06
(*E*)-2-Hexenal	53	47	65	35	0.1564	3.9695	0.6924	0.0463
(*Z*)-3-Hexenyl acetate	55	45	71	29	0.4350	7.9038	0.5095	0.0049
(*E*)-2-Hexenyl acetate	62	38	40	60	2.5261	1.7490	0.1120	0.1860

### Survival duration of *D. citri* adults

**Figure 1:**
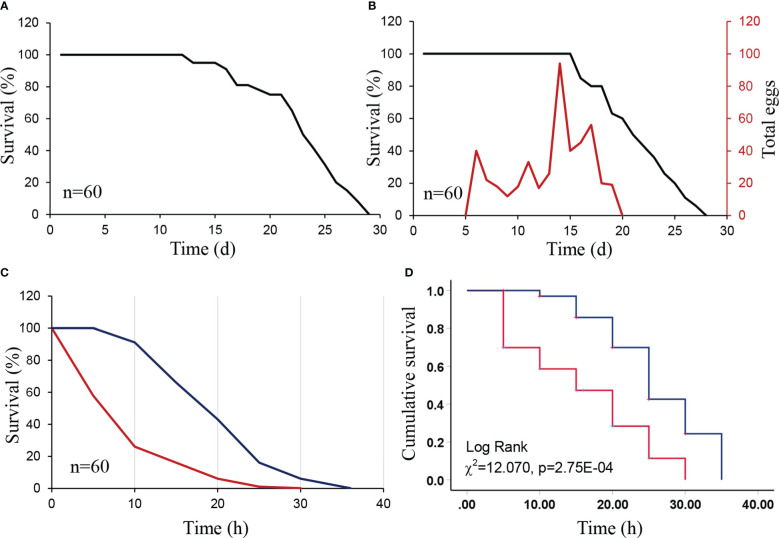
Survival curve and cumulative survival functions of *Diaphorina citri* adults over time when feeding on *Nicotiana tabacum* and a preferred host *Murraya exotica* (positive treatment). We also included a starvation treatment as a negative control. **(A)** Survival curve of male *D. citri* adults feeding on *M. exotica*. **(B)** Survival and fecundity curves of female *D. citri* adults feeding on *M. exotica*. **(C)** Survival curves of *D. citri* adults feeding on *N. tabacum* (red line) and starved (blue line). **(D)** Cumulative survival functions of *D. citri* adults on *N. tabacum* (red line) and the starved (control, blue line).

Survival of *D. citri* females and males on *M. exotica* was similar and less than 29 days, with high survival until 15 days of feeding (**Figures 1A, B** **)**. Moreover, females laid eggs on *M. exotica* leaves from days 5 to 20. The number of eggs laid on *M. exotica* peaked on day 15 (**Figure 1B**).

Survival of *D. citri* adults on *N. tabacum* lasted less than 30 h and was less than the starvation treatment (35 h) (**Figures 1C, D** **)**. There were significant differences in the survival of adult *D. citri* between *N. tabacum* and the starvation treatment (*χ* ^2^ = 12.070, *P* = 2.75E-04). In addition, the mean and median survival of adult *D. citri* on *N. tabacum* was 15.77 and 15 h, which are much shorter than those in the starvation treatment (25.976 and 25 h, respectively). Additionally, there were no eggs laid on *N. tabacum* leaves.

### *Diaphorina citri* feeding behavior on *N. tabacum*

**Figure 2:**
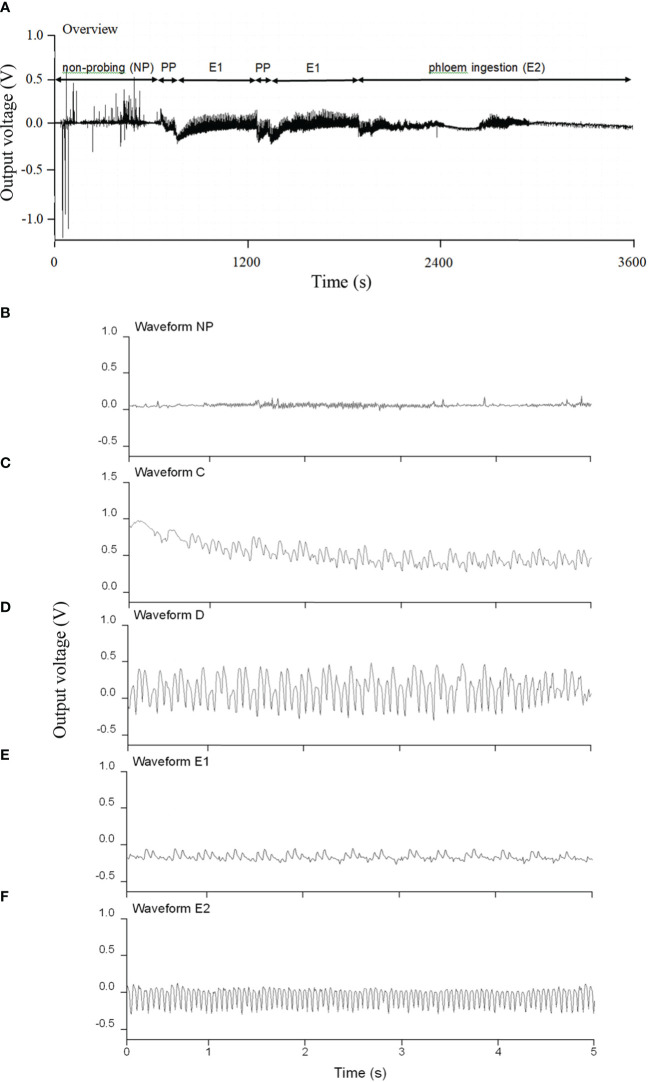
Typical electrical penetration graph (EPG) of an adult *Diaphorina citri* feeding on *Nicotiana tabacum* leaves. **(A)** EPG trace for the first 1 h after the start of assessment (NP = non-probing phase; PP = pathway phase (C + D), including intercellular probing of activity in the phloem **(C)** and phloem penetration **(D)**; E1 = phloem salivation; E2 = phloem ingestion). **(B)** Enlargement of waveform NP. **(C)** Enlargement of waveform C. **(D)** Enlargement of waveform D. **(E)** Enlargement of waveform E1. **(F)** Enlargement of waveform E2.

**Figure 3:**
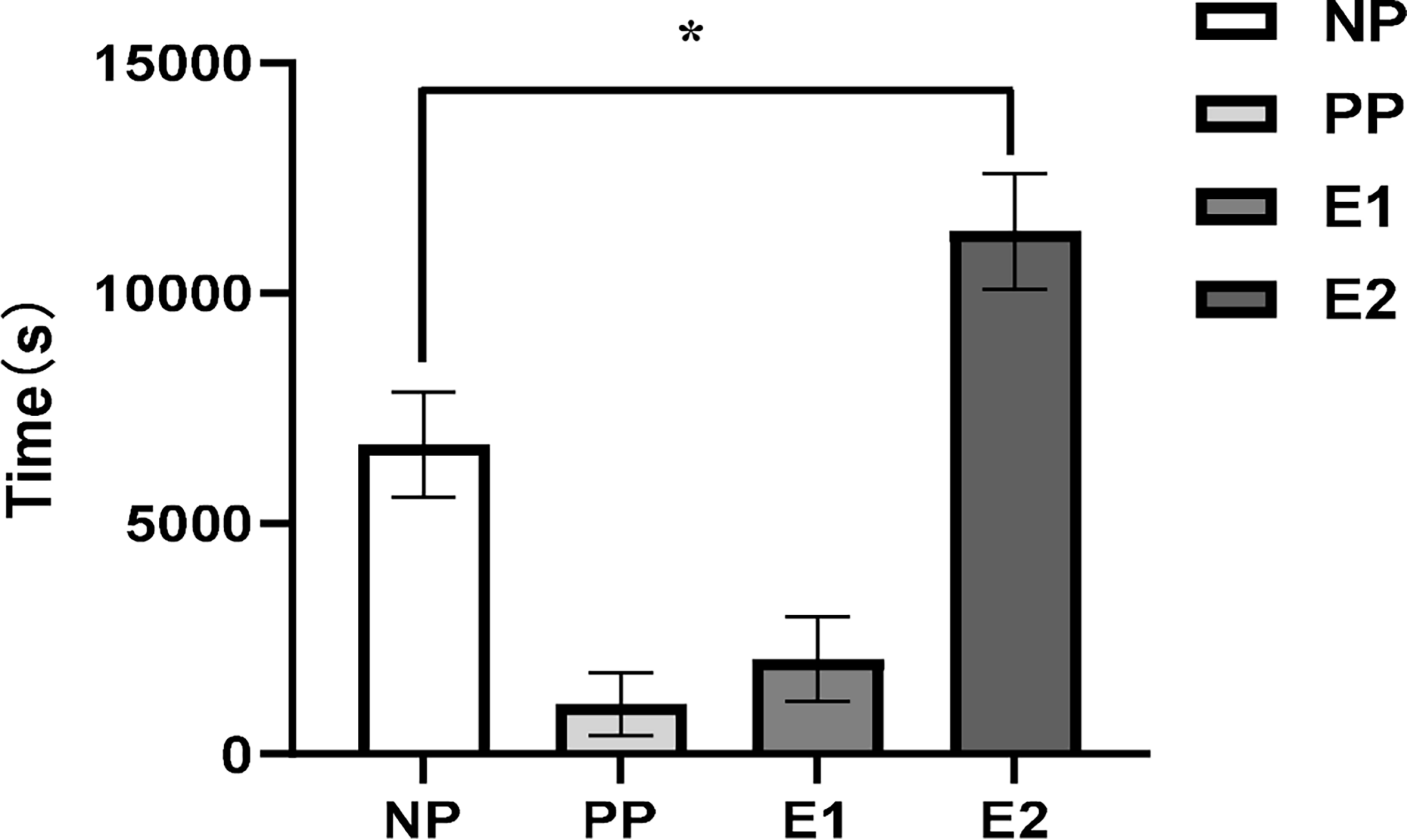
Mean ( ± SE, n = 5) duration of each feeding waveforms of adult *Diaphorina citri* feeding on *Nicotiana tabacum* leaves (ANOVA, LSD, *P < 0.05). (NP, non-probing phase; PP, pathway phase; E1, phloem salivation; E2, phloem ingestion).

**Figure 2** shows a representative EPG trace of the different feeding phases for *D. citri* on *N. tabacum* leaves. *Diaphorina citri* rarely had a waveform of xylem ingestion (G) on *N. tabacum*. The EPG waveform characteristics of pathway and phloem probing events on *N. tabacum*, involving the waveforms of NP (non-probing), PP (C and D), E1, and E2, are shown in **Figure 2**. Waveform C was the first waveform event, with an average amplitude of 40% and a frequency of 14–18 Hz. Waveform D possessed a lower frequency range of 3–7 Hz, whereas waveform E2 showed the highest frequency (14 Hz) (**Table 2**). As shown in **Figure 3**, during 6 h of feeding, the phloem ingestion phase (E2) was 52.5% of the total feeding time, and its duration was twice as long as the NP waveform (*P* = 0.000599), indicating that *D. citri* could feed on *N. tabacum* leaves.

**Table 2:** Main characteristics of *Diaphorina citri* electrical penetration graph (EPG) waveforms on tobacco leaves.

	Waveform characteristics		
EPG waveform	% Amplitude^1^	Frequency (Hz)	Main frequency (Hz)
C	40	14-18	15
D	–	3-7	4
E1	25	6-11	7.5
E2	10-45	9-14	14

^1^Medium amplitude; 1 V = 100% amplitude.

### Toxicity of L-nicotine on adult *D. citri*

**Figure 4:**
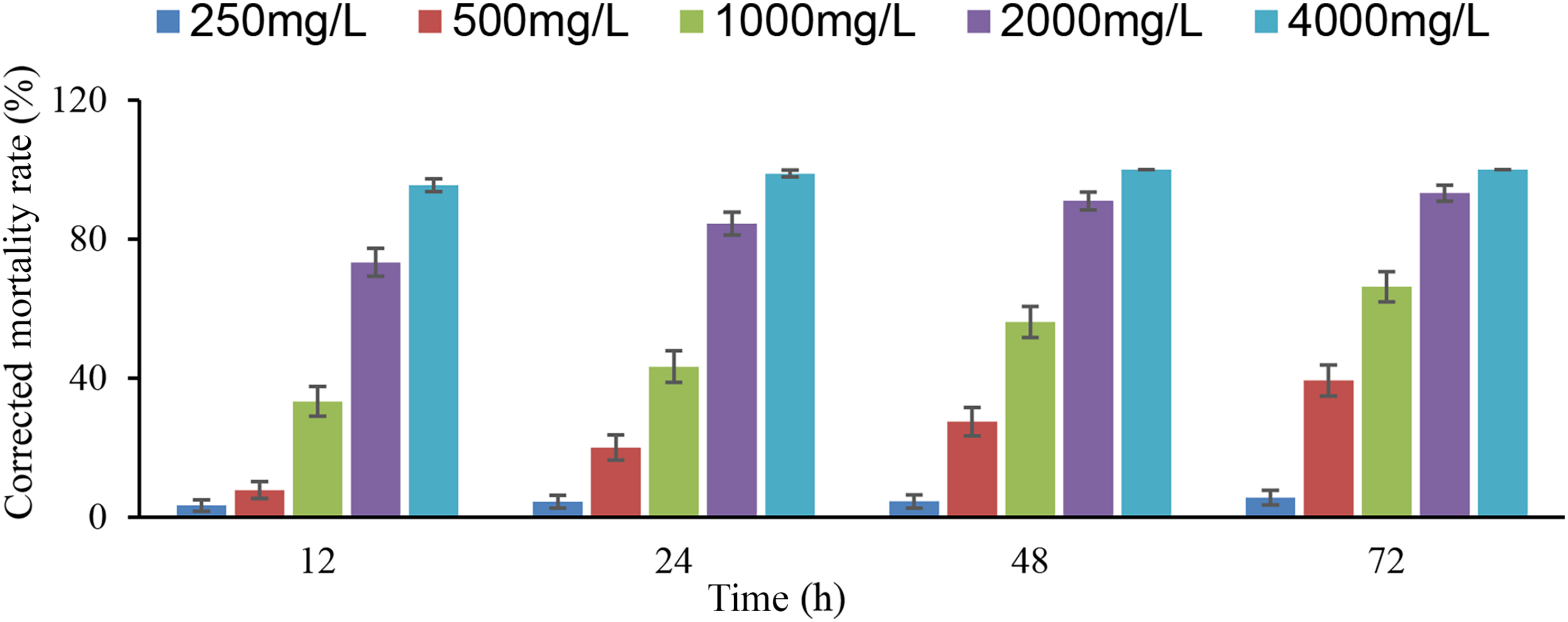
Corrected mortality rate of different concentrations of L-nicotine on *Diaphorina citri* adults.

The toxicity of L-nicotine on adult *D. citri* increased with increasing concentrations, as well as the treatment time (**Figure 4**). The corrected mortality rates of 2,000 and 4,000 mg/l were much higher than those of other concentrations, which was more than 70 and 95%, respectively. At the concentration of 1,000 mg/l, the corrected mortality rate (66.6%) at 72 h after exposure was twice than that of 12 h (33.3%). In addition, the LC_50_ for adult *D. citri* was 1,272 and 698 mg/l at 12 and 72 h after exposure, respectively. In general, the toxicity of L-nicotine on adult *D. citri* increased in time and was the highest at 72 h of exposure (**Table 3**).

**Table 3:** Toxicity of L-nicotine on adult *Diaphorina citri*.

Treatment time (h)	Linear regression equation*	χ^2^ (p)	LC_50_ (mg/L)	95% confidence interval (mg/L)
12	*y* = -9.677 + 3.117*x*	0.294 (0.961)	1,272	1,137-1,438
24	*y* = -9.380 + 3.136*x*	0.271 (0.965)	980	876-1097
48	*y* = -9.707 + 3.339*x*	0.401 (0.940)	806	721-901
72	*y* = -9.601 + 3.376*x*	0.212 (0.976)	698	622-780

*x_i_ = log_10_C_i_, MC_i_ = φ(z_i_), y_i_ = z_i_. C_i_ was the concentration, while MC_i_ was the corrected mortality rate.

## Discussion

Our study revealed that *N. tabacum* and synthetic GLVs (*Z*)-3-hexenol and (*E*)-2-hexenol are attractive to adult *D. citri*, supporting the notion that this insect relies on olfaction to locate its host plants. GLVs, especially leaf alcohols, play an important role in host location for other phytophagous insects, for example (*Z*)-3-hexen-1-ol and 1-hexanol for *Lygus rugulipennis* Poppius (Frati et al., 2008) and (±)-2-hexanol for *Aleurodicus dispersus* Russell (Zheng et al., 2014). (*E*)-2-hexenal and (*Z*)-3-hexenyl acetate, the synthetic GLVs from *N. tabacum*, were also attractive to male *D. citri* adults (**Table 3**), and (*Z*)-3-hexenyl acetate was reported to synergize with the sex pheromone of *H. armigera* (Kvedaras et al., 2007). Xia et al. (2017) found that *D. citri* adults prefer *N. tabacum* plants rather than Murcott tangor (host plant). In addition, previous studies have reported that *D. citri* adults may be able to use some non-host plants opportunistically as secondary host plants for acquiring water or alternate food when optimal hosts are scarce or absent (George et al., 2020; Lu et al., 2021). Thus, *D. citri* adults are likely to move from citrus orchards to *N. tabacum* if they grow next to each other.

Our EPG results showed that *D. citri* adults only secreted saliva and ingested sap from phloem in *N. tabacum* leaves and spent the longest duration in phloem sap ingestion (E2) (**Figure 2**), which is evidence of a phloem-feeding habit in this psyllid species (Bonani et al., 2010). *Diaphorina citri* adults performed more phloem feeding activities (D, E1, E2) than other stylet activities (NP, C) on *N. tabacum* leaves, which agree with observations that adults more readily feed on phloem from citrus to obtain nutrition (Bonani et al., 2010; Zhu et al., 2010). Moreover, CLas can occur in *N. tabacum* (Francischini et al., 2007), and Xia et al. (2017) reported that *D. citri* adults can also transfer the bacteria to *N. tabacum* after feeding. The survival time of *D. citri* on *N. tabacum* was much shorter than that under starvation conditions, suggesting that the death of adults on *N. tabacum* was not caused by starvation but could be related to the toxic components of *N. tabacum*. Nicotine is the predominant alkaloid accumulating in the leaves of most *N. tabacum* varieties and represents 90–95% of the total alkaloid content (Wang et al., 2000; Shoji et al., 2010). Several studies have demonstrated that neonicotinoid insecticides derived from nicotine, such as thiamethoxam and imidacloprid, are highly toxic to *D. citri* (Boina and Bloomquist, 2015; Miranda et al., 2016).

Previous studies have reported that *D. citri* may disperse from citrus orchards and utilize secondary host plants as a reservoir, facilitating their survival when primary host conditions are unfavorable or when these hosts are sprayed with insecticides (Lewis-Rosenblum et al., 2015; Johnston et al., 2018; Johnston et al., 2019; Lu et al., 2021). However, our study showed that *D. citri* adults that move to *N. tabacum* from citrus orchards would die after feeding on these secondary plants. Thus, *N. tabacum* could be used as a dead-end trap for adult *D. citri*.

For *N. tabacum* to kill adult *D. citri*, two conditions must be met. (i) *Nicotiana tabacum* should be grown nearby citrus crops. *Diaphorina citri* is critical for the spread of CLas due to their strong capacity to move between infected and healthy trees within an orchard (Wu et al., 2018). Insecticide applications to control *D. citri* are the primary management strategy currently recommended for CLas (Stansly et al., 2012) and could promote this movement. (ii) *Diaphorina citri* adults must be pushed away from citrus trees by insecticide applications or as a result of poor host conditions. Basically, adult *D. citri* dispersal requires citrus trees to either lack citrus flushes or to be sprayed with pesticides. However, while moving to *N. tabacum*, *D. citri* are unable to survive (**Figure 1**, Xie et al., 2015; Xia et al., 2017). In this context, *N. tabacum* could be used as a dead-end trap plant, which has considerable potential in integrated pest management (IPM) for *D. citri*. Several kinds of dead-end trap plants have been suggested and used in IPM, such as *N. tabacum* and *Barbarea vulgaris* R. Br. for *Plutella xylostella* (L.) larvae (Gupta and Thorsteinson, 1960; Shelton and Nault, 2004; Møldrup et al., 2012) and *Vetiveria zizanioides* (L.) Nash for *Chilo suppressalis* Walker larvae (Lu et al., 2019).

*Diaphorina citri* eggs were only observed on *M. exotica* plants (**Figure 1B**). The pre-oviposition period of *D. citri* females ranged from 7 to 20 days (Li et al., 2019); thus, it is likely that females on *N. tabacum* died before laying eggs. Furthermore, adults on *N. tabacum* died within 30 h of feeding, whereas they initiated oviposition 5 days after feeding on *M. exotica*. In other words, *N. tabacum* is very effective as a dead-end trap because *D. citri* females were attracted to the plants, fed on them, and died shortly prior to laying any eggs. Our results suggest that it is worthwhile to grow *N. tabacum* in and around citrus orchards. To optimize area-wide management of *D. citri*, field studies are needed to evaluate the use of *N. tabacum* surrounding citrus orchards as dead-end traps.

## Data availability statement

The original contributions presented in the study are included in the article/**Supplementary Material**, further inquiries can be directed to the corresponding authors.

## Author contributions

LZ, WW, and WC designed the study. LZ performed the majority of experiments with the help of QX, GG, and YL. GG and WW analyzed the data, and LZ wrote the first version of the manuscript. MY, SS, and WC revised the manuscript and approved the submitted version. All authors contributed to the article and approved the submitted version.
