# Supplementary material for: Nicotiana tabacum as a dead-end trap for adult Diaphorina citri: A potential biological tactic for protecting citrus orchards
Source: Front Plant Sci. 2023 Jan 6;13:1081663. doi: 10.3389/fpls.2022.1081663 (PMC9853912; doi:10.3389/fpls.2022.1081663)
Supplement: Supplementary file 1 [file DataSheet_1.docx]

**SUPPLEMENTARY FIGURES**


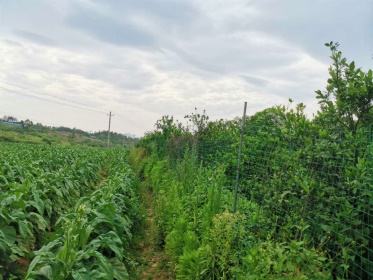

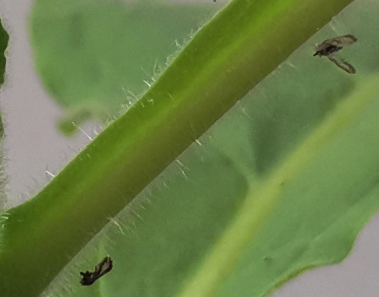


**(A)**

**(B)**

**FIGURE 1.** *Diaphorina citri* adults moving from the citrus orchards died on tobacco leaves. (**A**) The tobacco field around a citrus orchard; (**B**) The dead *D. citri* adults on tobacco leaves.


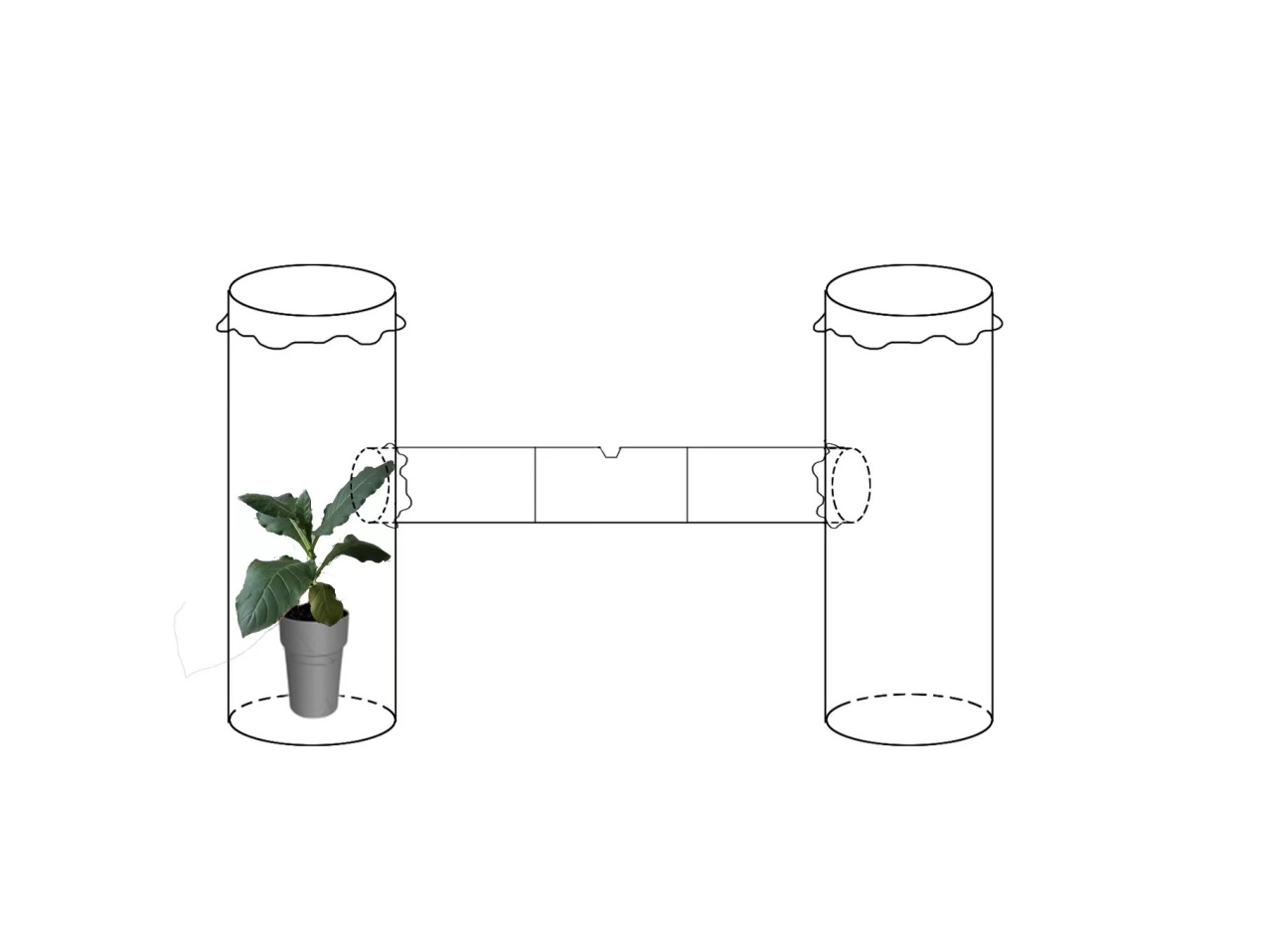


1

2

3

4

5

6

**FIGURE 2** Schematic diagram of the H-tube olfactometer (1: gauze; 2: no-choice area; 3: entrance; 4: choice area; 5: tobacco plant; 6: cylinder (arm)).


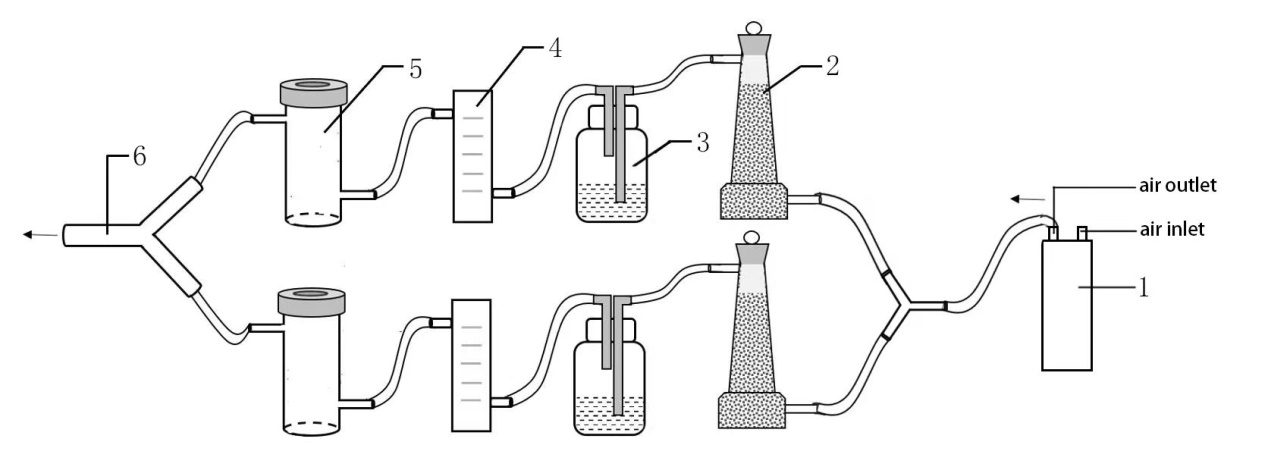


**FIGURE 3** Schematic diagram of the Y-tube olfactometer (1: atmospheric sample collector; 2: drying tower; 3: humidifier; 4: flowmeter; 5: odor source bottle; 6: Y-tube; the arrows showing the airflow direction from the atmospheric sample collector to the Y-tube).


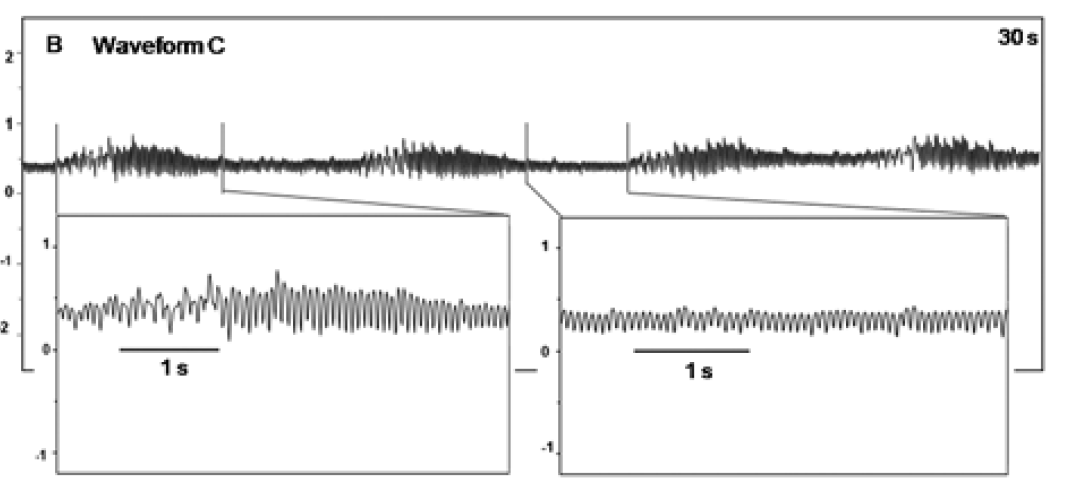

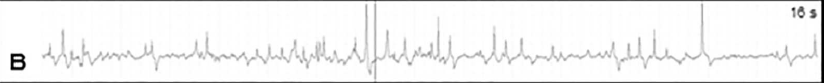


Waveform NP


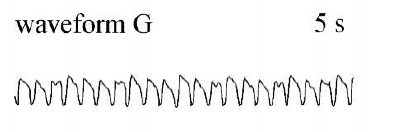

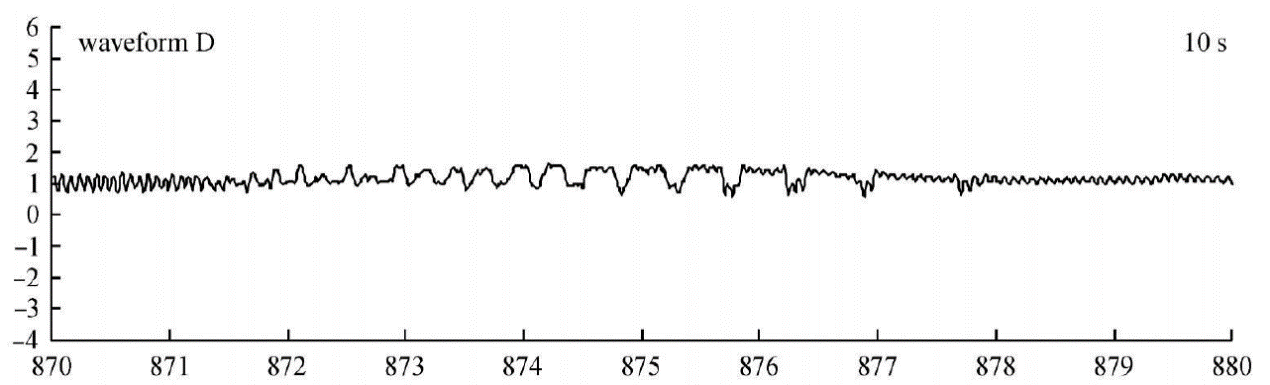


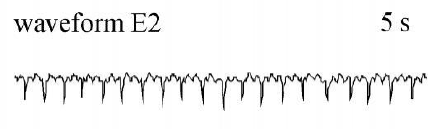

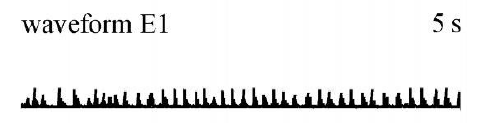


**FIGURE 4** EPG waveforms of Asian citrus psyllid from Bonani et al., 2010; Yang et al., 2011; George et al., 2017. Waveform NP represents non-probing phase; Waveform C is complex, showing extracellular voltage level and significant oscillations in frequency (11.5-19 Hz), and is the first waveform event observed in all probes; Waveform D is always observed between waveforms C and E1, with the trait of a frequency of 1-6 Hz and extracellular voltage level; Waveform E1 always started with a potential drop after waveform D, showing a frequency range of 5-10 Hz, and negative voltage level indicated intracellular activity; Waveform E2 (a frequency of 4-9 Hz) is always preceded by E1, showing intracellular voltage level, and can last for a long time; Waveform G exhibits the same extracellular voltage level as waveform C, and has the biggest amplitude among all waveforms, with a frequency of 7 Hz association with xylem sap ingestion.
